# Degrading traumatic memories with eye movements: a pilot functional MRI study in PTSD

**DOI:** 10.3402/ejpt.v7.31371

**Published:** 2016-11-29

**Authors:** Kathleen Thomaes, Iris M. Engelhard, Marit Sijbrandij, Danielle C. Cath, Odile A. Van den Heuvel

**Affiliations:** 1 Department of Psychiatry, GGZinGeest/VU University Medical Center, Amsterdam, The Netherlands; 2 Department of Clinical Psychology, Utrecht University, Utrecht, The Netherlands; 3 Department of Clinical and Developmental Psychology, VU University, Amsterdam, The Netherlands; 4 Altrecht Academic Anxiety Center, Utrecht, The Netherlands; 5 Department of Anatomy & Neurosciences, VU University Medical Center, Amsterdam, The Netherlands

**Keywords:** Posttraumatic stress disorder, eye movement desensitization and reprocessing, working memory, amygdala, functional MRI

## Abstract

**Background:**

Eye movement desensitization and reprocessing (EMDR) is an effective treatment for posttraumatic stress disorder (PTSD). During EMDR, the patient recalls traumatic memories while making eye movements (EMs). Making EMs during recall is associated with decreased vividness and emotionality of traumatic memories, but the underlying mechanism has been unclear. Recent studies support a “working-memory” (WM) theory, which states that the two tasks (recall and EMs) compete for limited capacity of WM resources. However, prior research has mainly relied on self-report measures.

**Methods:**

Using functional magnetic resonance imaging, we tested whether “recall with EMs,” relative to a “recall-only” control condition, was associated with reduced activity of primary visual and emotional processing brain regions, associated with vividness and emotionality respectively, and increased activity of the dorsolateral prefrontal cortex (DLPFC), associated with working memory. We used a randomized, controlled, crossover experimental design in eight adult patients with a primary diagnosis of PTSD. A script-driven imagery (SDI) procedure was used to measure responsiveness to an audio-script depicting the participant's traumatic memory before and after conditions.

**Results:**

SDI activated mainly emotional processing-related brain regions (anterior insula, rostral anterior cingulate cortex (ACC), and dorsomedial prefrontal cortex), WM-related (DLPFC), and visual (association) brain regions before both conditions. Although predicted pre- to post-test decrease in amygdala activation after “recall with EMs” was not significant, SDI activated less right amygdala and rostral ACC activity after “recall with EMs” compared to post-“recall-only.” Furthermore, functional connectivity from the right amygdala to the rostral ACC was decreased after “recall with EMs” compared with after “recall-only.”

**Conclusions:**

These preliminary results in a small sample suggest that making EMs during recall, which is part of the regular EMDR treatment protocol, might reduce activity and connectivity in emotional processing-related areas. This study warrants replication in a larger sample.

**Highlights of the article:**

- Script driven imagery (SDI) before and after recall of traumatic memories is feasible to investigate working mechanisms of degrading of traumatic memories with eye movements (EMs) in PTSD.
- Right amygdala and rostral ACC activity was significantly lower after “recall with EMs” than after “recall-only”.
- Functional connectivity from amygdala to rostral ACC was decreased after “recall with EMs” vs. “recall-only”.
- This study warrants replication in a larger sample.

About 9–18% of trauma-exposed persons suffer from posttraumatic stress disorder (PTSD), which involves considerable impairments in functioning (Breslau et al., 1998). Hallmark symptoms of PTSD are intrusive, traumatic memories with intense emotionality and vividness that interfere with daily life (e.g., Boe, Holgersen, & Holen, 2010). According to the (inter) national clinical guidelines based on meta-analyses, the most effective treatments for PTSD are trauma-focused cognitive behavioral treatment, such as prolonged exposure (PE) and cognitive therapy (CT), and Eye movement desensitization and reprocessing (EMDR) (Van Balkom et al., 2013; World Health Organization, 2013). Traumatic memories are the main target of these treatments. PE stimulates a habituation process and corrective learning through repeated imagine and *in vivo* exposure exercises, while CT mainly consists of repeatedly challenging maladaptive cognitions that developed following the traumatic event, through the use of cognitive restructuring techniques (Resick, Nishith, Weaver, Astin, & Feuer, 2002). A crucial component of EMDR is that the patient recalls traumatic memories, particularly images with high emotional load (“hot spots”), while simultaneously making horizontal eye movements (EMs) induced by the therapist's finger moving across the patient's visual field. A meta-analysis has shown that these EMs add to EMDR's effectiveness, although standardized laboratory studies (*N*=10) in healthy persons showed moderate to large effect sizes (0.74), while effect sizes in clinical studies (*N*=24) were small (0.27–0.41) (Lee & Cuijpers, 2013).

It has been unclear why performing EMs is effective. The first “adaptive information processing” explanation hypothesized that EMDR stimulates inter-hemispheric communication (Shapiro, 1989). This mechanism, however, was not supported by two electroencephalography (EEG) studies, showing no or *decreased* functional inter-hemispheric interaction after EMDR (Propper, Pierce, Geisler, Christman, & Bellorado, 2007; Samara, Elzinga, Slagter, & Nieuwenhuis, 2011). A second “orienting response” explanation gained some empirical support (Barrowcliff, Gray, MacCulloch, Freeman, & MacCulloch, 2003; Sack, Lempa, Steinmetz, Lamprecht, & Hofmann, 2008; Schubert, Lee, & Drummond, 2011). According to this explanation, EMs induce heightened alertness, enhancing exploratory behavior in which cognitive processes become more flexible and efficient. This arousal phase is thought to be followed by a reflexive de-arousal phase. Some physiological changes associated with the EMs fit with the orienting response hypothesis, such as changes in skin conductance and heart rate (Elofsson, von Schèele, Theorell, & Söndergaard, 2008; Sack et al., 2008; Schubert et al., 2011). However, other changes, such as increased respiration, are not consistent with the proposed mechanism of action (Schubert et al., 2011). The third explanation, based on a “working memory (WM) theory,” states that the two tasks (recall and EMs) compete for limited capacity WM resources (see Gunter & Bodner, 2008; Van den Hout & Engelhard, 2012). According to this theory, EMs render memories more labile during recall, and competition of the EMs and the recall for the limited capacity of the WM, leads to the degrading of visual images in respect of vividness and emotionality. This memory degrading is thought to persist upon future recalls, because memory recall is affected by the nature of earlier recalls (Baddeley & Andrade, 2000; Van den Hout & Engelhard, 2012). Laboratory studies have been performed in healthy persons who recall an aversive memory while making EMs (“recall with EMs”) or without making EMs (“recall-only”) in a crossover design. These studies have demonstrated that “recall with EMs” reduces vividness and emotionality of the aversive memories upon later recall up to 1 week later (e.g., Engelhard, Van den Hout, & Smeets, 2011; Gunter & Bodner, 2008; for review, see Van den Hout & Engelhard, 2012). These findings have been replicated in PTSD patients (Van den Hout et al., 2012). The WM theory is further supported by, for instance, the findings that (1) other WM taxing dual-tasks are also effective, such as counting backwards or playing Tetris (e.g., Engelhard, Van Uijen, & Van den Hout, 2010; Engelhard et al., 2011), and passive tasks (e.g., listening to tones) are less effective (Van den Hout et al., 2011, 2012), and (2) there is a dose-response relationship between WM load and its effects (Engelhard et al., 2011; Gunter & Bodner, 2008).

However, most studies relied on self-report ratings of vividness and emotional intensity (see Kearns & Engelhard, 2015), which may be prone to demand bias and self-representation strategies. Neurobiological research might aid in this knowledge gap, if focused on brain areas involved in WM, that is, frontoparietal areas such as the bilateral dorsolateral prefrontal cortex (DLPFC) and left ventrolateral PFC (Curtis & D'Esposito, 2003; Owen, McMillan, Laird, & Bullmore, 2005; Rottschy et al., 2012), and brain areas involved in visual recall, such as the visual (association) cortices (Ganis, Thompson, & Kosslyn, 2004; Kosslyn, Ganis, & Thompson, 2001), and emotional processing areas, such as the amygdala, anterior insula, rostral anterior cingulate cortex (ACC), and dorsomedial prefrontal cortex (DMPFC) (Rabinak et al., 2014; Rauch, Shin, & Phelps, 2006; Shin et al., 2004).

So far, neurobiological studies on EMDR are sparse. In a functional magnetic resonance imaging (fMRI) study in 22 healthy controls, it was found that bilateral alternating auditory stimulation while viewing disgusting pictures resulted in increased amygdala activity and decreased DLPFC activity (Herkt et al., 2014). In patients, no such experimental studies yet exist. There are, however, some patient studies using EEG before and after EMDR treatment. One study using a modified oddball paradigm containing auditory standard, target, and novel tones, showed decreased P3a component of event-related potentials after EMDR, suggesting reduced alertness and de-arousal upon treatment (Lamprecht et al., 2004). Two other EEG studies from the same lab, showed a shift from the medial prefrontal cortex and fronto-temporal activity *during EMs* in the first EMDR session, toward more posterior associative regions in the last session, that correlated with symptom improvement (Pagani et al., 2015; Trentini et al., 2015). However, these EEG studies recorded brain activity only *during* EMs and not after the intervention when—if successful—the memories lost vividness and emotionality. As far as we know, no studies have yet examined the brain responds to memory recall of the—degraded—traumatic memory after an EM intervention.

The general aim of the present study was to gain insight into neurobiological mechanisms underlying the effects of EMDR. First, we investigated if recall of traumatic memories after recall while simultaneously making EMs (“recall with EMs”) is associated with increased activity in brain regions associated with WM, such as the DLPFC, as compared to activity after a recall only control condition (“recall-only”). Second, we investigated if “recall with EMs,” compared to “recall-only,” is also associated with decreased activity in visual brain regions areas and in brain regions associated with emotional processing, specifically the amygdala, insula, rostral ACC, and DMPFC, as compared to “recall-only.” Third, we explored whether functional connectivity between these areas (DLPFC, visual cortex, amygdala, insula, rostral ACC, and DMPFC) is differentially modulated by “recall with EMs” and “recall-only.” We hypothesized that if “recall with EMs,” relative to “recall-only,” degrades traumatic memories, that is, decreasing their vividness and emotionality via a WM mechanism, this would be associated with reduced visual (association) cortex activation, reduced amygdala, insula, rostral ACC, and DMPFC activity and increased DLPFC activity during memory recall after the intervention.

## Methods

### Subjects

PTSD patients included in the study were seeking treatment at the specialized Altrecht Academic Anxiety Center in Utrecht or at the Primary Mental Health Care Center Prezens in Amsterdam. Patients with repeated sexual or physical abuse (type II trauma), current psychotic or substance use disorder, disturbing medical conditions, cardiovascular medication, contra-indications for MRI (metal implants, pregnancy, and claustrophobia), and previous EMDR sessions were excluded. From psychotropic medication only 3-months-stabile selective serotonin reuptake inhibitors (SSRIs) were accepted. In Altrecht, 47 patients were asked for participation, from whom 34 were excluded (10 complex trauma and/or comorbid personality problems, 10 refusals, four previous EMDR, three low IQ, two non-responses, one insufficient fluency in Dutch, one no PTSD, one psychosis, one pregnant, and one pacemaker) and 13 were scanned (26%). In Prezens, 37 patients were invited for participation by their therapists, of whom only two participated in the study, possibly because of reorganization of the mental healthcare program at the time of the study. From these 15 scan sessions, seven were not useful (two interrupted by the patient due to feelings of panic, three lost because of moving artifacts, and two lost because of technical problems) resulting in eight useful series of MRI scans. The Medical Ethical Committee of the VU University Medical Center, Amsterdam, approved the study. Written informed consent was obtained from each participant.

### Outcome measures

PTSD diagnosis, psychiatric disorders other than PTSD, and trauma history were assessed using the Structured Clinical Interview for DSM-IV Axis-I disorders (First, Spitzer, Williams, & Gibbon, 1995). PTSD symptoms were assessed with the Posttraumatic Symptom Scale—Self Report (PSS-SR; Engelhard, Arntz, & Van den Hout, 2007; Foa, Riggs, Dancu, & Rothbaum, 1993). The PSS-SR contains 17 items corresponding to the DSM-IV symptoms of PTSD ranging from 0 (not at all) to 3 (almost always) and scores range from 0 to 51. State anxiety was assessed with the state subscale of the State-Trait Anxiety Inventory (Spielberger, 1983; Van der Ploeg, Defares, & Spielberger, 1980), consisting of 20 items for state anxiety. All items are rated on a 4-point scale from 1 (not at all) to 4 (very much), resulting in a range of 20–80. Finally, the 21-item Beck Depression Inventory (BDI) was administered, with scores from 0 (not at all) to 3 (very much), ranging from 0 to 63 (Beck, Steer, & Garbin, 1998). Directly after the scan session, the clinician-administered dissociative states scale (CADSS, Bremner et al., 1998) was administered (28 items, scores from 0 (not at all) to 4 (extreme), range 0–112) to rule out dissociative states during scanning.

The main outcome measure was neural activation, as measured by blood-oxygen-level-dependent (BOLD) responses, in response to memory recall during script-driven imagery (SDI). We used the SDI procedure to compare changes in brain responses during memory retrieval of the traumatic memory (before versus after recall) for the 2 experimental conditions, that is, “recall with EMs” and “recall-only.” This well-established procedure has been used in numerous studies of PTSD patients, has shown to produce highly reliable psychophysiological and neurobiological responses (e.g., Orr & Roth, 2000; Pitman, Orr, Forgue, De Jong, & Claiborn, 1987), has recently been used in a study of the effects of EMs (Kearns & Engelhard, 2015), and has been adapted for fMRI (Lanius et al., 2002). To prepare for the SDI, the participant had a short interview (15 min) to identify two hot spots of one personal traumatic event with comparable vividness and emotionality. These two hot spots were randomly assigned to the labels “script 1” and “script 2.” The executive researcher (KT) read each of the two selected hot spots aloud and made an audio recording of 30 s each, to play back during scanning (cf. Pitman et al., 1987). SDI procedure consists of four phases: baseline (60 s), listening to the personalized audio-script (30 s), imagining the traumatic event (30 s), and recovery (60 s) (see next paragraph for a detailed description of the phases).

### Experimental procedure in the scanner

The MRI experiment was set up as a randomized, controlled, crossover (within-subject) design (see Supplementary Fig. 1). Scripts 1 and 2 were randomized to the “recall with EMs” or “recall-only” condition, and the order of the conditions was randomized as well. During “recall with EMs” participants were asked to recall the traumatic image during eight blocks of 24-s, with a 10-s break in between, and simultaneously to visually track a 1-cm dot moving from one side of the screen across to the other side at a rate of one movement per second while instructed to continue recalling the traumatic image (cf. the procedure by Engelhard et al., 2010). In the “recall-only” condition, patients were asked to recall the other traumatic image while keeping their eyes open and look around on the screen where a stationary dot was projected. All participants performed both the “recall with EMs” and “recall-only” conditions with a structural MRI (sMRI) (8 min) in between. During the sMRI, patients did a short distraction task (“Think of words starting with an A”) to prevent to return to any memories from the previous condition.

Before and after the “recall with EMs” and “recall-only” conditions, SDI was carried out in the scanner. The SDI procedure consists of four phases: baseline (60 s), listening to the personalized audio-script (30 s), imagining the traumatic event (30 s), and recovery (60 s). The baseline instruction (“Just lay still, breath quietly. You don't have to do anything yet”) was showed on a screen seen through a mirror mounted above the subject's head. Then the audio-script was presented through headphones with the instruction “Listen carefully to the script” on the screen. Upon hearing the script, the subject was encouraged to imagine as if the traumatic event occurred again by remembering visual, olfactory, auditory, and somatosensory sensations associated with the traumatic event (instruction presented on the screen: “Continue to hold the image. What do you see? What do you smell? What do you hear? What do you feel?”). Then, during the recovery phase subjects were asked to lay still (instruction on the screen: “Let go of the image. Breathe quietly in and out”). All four phases were repeated once.

### MRI scanner

The MRI scan session took place at the Spinoza Centre for Neuroimaging, Amsterdam, on a Philips Achieva XT 3T 32 channel MR system and analyzed using SPM8. The MRI protocol consisted of a survey scan to check the field of view (FOV), one fMRI scan for each condition, that is, scans sensitive to local changes in blood oxygenation level (T2 single shot GE-EPI sequence (MS-FFE); TR/TE=2,000/27.63 ms, FA=76.1, FOV=240×2 mm, voxel size=3×3 mm, slice gap 0.3) and an sMRI scan for a high-resolution anatomical image (T1-weighted scan, TR/TE=8.2/3.8 s, FA=8, 220 slices, FOV=204×188, voxel size=1×1×1 mm). Stimuli were presented using the E-Prime 2.0 software (Psychology Software Tools, Pittsburgh, PA). A mirror attached to the head coil allows subjects to comfortably view the task while lying in the MRI scanner. Performance of EMs was checked during fMRI with an Eyelink-1000 eye-tracker, with fiber optic camera upgrade (SR Research Ltd., Mississauga, Ontario, Canada). Audio-scripts were presented through MR-compatible headphones (MR confon sound system, GmbH, Leibnitz-Institute for Neurobiology, Magdeburg, Germany). All subjects had normal or corrected-to-normal vision (by using MRI-compatible glasses) and normal hearing. We checked if arousal, as measured by subjective general distress levels (researcher asked through headphone: “How tense are you right now? With 0=not tense at all, and 100=extremely tense”) and heart rate, before the “recall with EMs” and “recall-only” conditions was similar, in order to ensure that arousal returned back to the starting point between conditions. In addition, before and after each presentation of the script, subjects scored subjective vividness and emotionality of the traumatic memory on a 0–100 VAS scale (0=not vivid at all—100=extremely vivid, resp. 0=not unpleasant at all—100 extremely unpleasant), using an MRI-compatible button box (HHSC-2×4-C) to a projected VAS-scale on the screen.

### Demographical and clinical data analyses

Mean, median, standard deviation, minimum, and maximum were calculated for all demographical and clinical data with IBM SPSS Statistics Version 22.0.0.0. Subjective ratings or emotionality and vividness were analyzed using repeated measure ANOVA's with the factors Time (pre–post) and Condition (“recall with EMs”—“recall-only”) to test for main effects and interactions.

### MRI analyses

fMRI data were preprocessed and analyzed using the software package SPM8 (Wellcome Department of Imaging Neuroscience, London, UK), implemented in Matlab (The Mathworks, Inc., Natick, MA). We performed standard preprocessing steps, including manual reorientation to the anterior commissure, realignment and unwarping, coregistration of the mean image to the structural (T1-weighted) MR image, spatial normalization into the Montreal Neurological Institute (MNI) standard space, reslicing to 3×3×3 mm voxels, and spatial smoothing with “an 8-mm FWHM (full-width-at-half-maximum) Gaussian filter” Task-related change in brain activity in response to trauma recall was measured as the change in the BOLD-response (whole brain) during the imagine phases compared to the baseline phases from the SDI procedure (Imagine-versus-baseline contrast). Because all SDI phases were repeated once, brain activation was calculated as the mean activation pattern of these phases.

For the first-level processing SPM-model, we calculated four Imagine-versus-baseline contrasts: (1) before the moving dot, that is, pre-“recall with EMs,” (2) after the moving dot, that is, post-“recall with Ems,” (3) before the steady dot, that is, pre-“recall-only,” and (4) after the steady-dot, that is, post-“recall-only.” First-level contrast images containing parameter estimates of these contrasts were entered into a 2nd-level analysis comparing time (pre−post) and conditions (“recall with EMs” and “recall-only”). A high-pass filter (128 s cutoff period) and movement parameters were used to remove noise associated with low frequency confounds and control for moving confounds. Applying regions-of-interest (ROI) analyses, we used an explicit mask created with Marsbar, consisting of the WFU-pickatlas 3D bilateral mask of the amygdala, and 10-mm-radius spheres around the MNI coordinates of the DLPFC (MNI: 44, 34, 32 & −46, 26, 24), rostral ACC (MNI: 10, 47, 10 & −10, 41, 5), DMPFC (MNI: 1, 25, 46 & −1, 22, 51), anterior insula (MNI: 36, 22, −6 & −32, 22, −2) and visual cortex (MNI: 5, −86, 9 & −12, −81, 14), based on recent neurobiological review papers on WM (Rottschy et al., 2012), SDI (Rabinak et al., 2014), and visual imagery (Ganis et al., 2004).

Exploratory functional connectivity analyses were conducted on task-related fMRI data, using generalized psychophysiological interactions (gPPI) (McLaren, Ries, Xu, & Johnson, 2012), with the bilateral amygdala as seed, to the ROIs (bilateral DLPFC, rostral ACC, DMPFC, anterior insula, and visual (association) cortex). The physiological variable was created by extracting the mean deconvolved time course from the seed region. Psycho-physiological (PPI) terms were computed as the cross product of the physiological variable and each task regressor (pre-“recall with EMs,” post-“recall with EMs,” pre-“recall-only,” and post-“recall-only”). This resulted in nine regressors: four task conditions, four PPI terms, and the time course of one seed region. Contrasts between the PPI at each condition were brought to 2nd level in a full-factorial model. Performance was added as covariate of no interest.

All fMRI analyses above the threshold for a statistical significance of *p*-uncorrected<0.001 (*Z*≥3.09) and cluster size threshold of *k*≥5 were reported (Cox, 1996), and we indicated where *p*-family-wise-error (*p*-FWE)-corrected values for multiple comparisons were below 0.05 (*).

## Results

### Subjects

Subjects were eight right-handed patients (five women and three men), primarily diagnosed with PTSD (DSM-IV) after a single traumatic event (three assault, three motor vehicle accidents, and three intimate partner violence), recruited via the Academic Anxiety Center Altrecht (Utrecht) and Primary Mental Health Care (Prezens, Amsterdam). Mean age was 37.4 years (SD 8.1). Mean duration of education was 11.3 (SD 3.0) years. Psychiatric comorbidity consisted of a major depressive disorder (*n*=7), periodic explosive disorder (*n*=1), panic disorder (*n*=1), or social phobia (*n*=1). One patient used a stable dose citalopram 20 mg. Mean severity of PTSD was 32.5 (SD 7.2), and depressive symptoms were severe (mean BDI: 31.6, SD 6.8). State dissociation (CADSS) during the scan session was minimal (mean 3.5, SD 3.7). Heart rate pre-“recall with EMs” did not differ significantly from pre-“recall-only” (79.0 (11.0) bpm versus 75.0 (8.6) bpm, *t*=2.1, *p*=0.08), indicating that arousal before conditions was similar.

### Subjective levels of general distress, vividness and emotionality

Table 1 shows that mean general level of distress was higher after the “recall with EMs” condition as compared with the “recall-only” condition (rmANOVA: Time, *F*=2.6, *p*=0.16, Condition *F*=11.3, *p*=0.02; or Time×Condition *F*=0.5, *p*=0.51). Subjective vividness did not change significantly over time (rmANOVA: Time *F*=0.1, *p*=0.74, Condition *F*=1.6, *p*=0.25, Time×Condition *F*=0.6, *p*=0.45). Subjective emotionality also did not change significantly over time (rmANOVA: Time *F*=1.1, *p*=0.32, Condition *F<*0.1, *p*=0.85, Time×Condition *F*=1.0, *p*=0.36).

**Table 1:** Subjective levels of general distress, vividness and emotionality during the scan session in PTSD patients

	Pre-“recall with EMs”	Post-“recall with EMs”	Pre- “recall-only”	Post-“recall-only”	Time (*F*, *p*)	Condition (*F*, *p*)	Time×condition (*F*, *p*)
General distress^a^	27.1 (17.0)	41.4 (23.4)	20.0 (14.1)	25.7 (24.4)	2.6, 0.16	11.3, 0.02*	0.5, 0.51
Vividness^b^	72.8 (15.7)	73.8 (8.7)	80.5 (9.2)	77.5 (13.0)	0.1, 0.74	1.6, 0.25	0.6, 0.45
Emotionality^c^	76.4 (13.6)	76.1 (9.8)	79.8 (9.2)	74.8 (12.6)	1.1, 0.32	0.1, 0.85	1.0, 0.36

EMs, eye movements.

a General distress on a VAS scale “How tense are you right now?” With 0=not tense at all—100=extremely tense;

b subjective vividness of the traumatic memory on a VAS scale (0=not vivid at all—100=extremely vivid);

c subjective emotionality of the traumatic memory on a VAS scale (0=not unpleasant at all—100 extremely unpleasant).

* *p<*0.05.

### Imaging results

**Fig 1:**
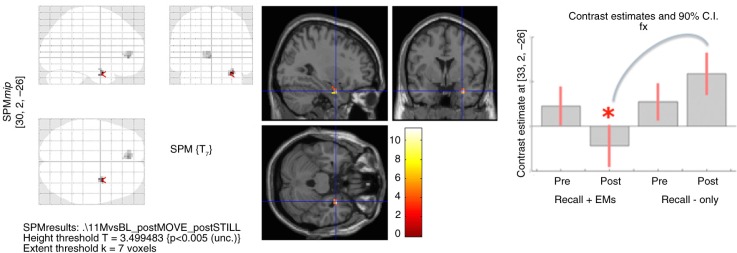
Full-factorial analysis of the script-driven imagery (see Table 2), showing on the left panel: the glass brain with decreased right amygdala and rostral ACC activity post-“recall with EMs” compared with post-“recall-only”; on the middle panel: *T*-values of decreased right amygdala activity; and on the right panel: contrast estimates in the right amygdala per time and condition: decreased right amygdala activity post-“recall with EMs” compared with post-“recall-only.” Applying regions-of-interest (ROI) analyses, we used an explicit mask, consisting of the bilateral mask of the amygdala, DLPFC, rostral ACC, DMPFC, anterior insula, and visual cortex (see Methods for detailed information). Images were set at a threshold of *p*-uncorrected <0.005 for multiple comparisons and *k*≥5 for illustrative purposes.

In the Imagine-versus-baseline contrast of all four conditions together (pre- and post-“recall with EMs” and “recall-only”), patients showed increased activity in the bilateral visual (association) cortex, left DLPFC, DMPFC, and right anterior insula (see first row of Table 2). The full-factorial analysis showed no significant main effect for Time or Condition and a non-significant interaction effect of Time×Condition in the right amygdala (see Table 2). However, *post-hoc t*-tests showed that right amygdala and rostral ACC activity was significantly lower after “recall with EMs” compared with post-“recall-only” (Fig. 1). *Post-hoc t*-tests showed no significant differences between the pre-“recall with EMs” and pre-“recall-only” conditions. We found no interaction or effects of Time×Condition in the DLPFC.

**Table 2:** Brain activity (BOLD responses) in the Imagine-versus-baseline contrast from regions-of-interest (ROI) analyses, with an explicit bilateral mask of the amygdala, DLPFC, rostral ACC, DMPFC, anterior insula, and visual cortex

	Imagine-versus-baseline contrast	MNI	*K*	*Z*	*p*-FWE-corrected for multiple comparisons
Main task effect of SDI					
All conditions	R visual cortex (BA17/18)	15,−97,10	52	4.99	0.000**
	L visual cortex (BA17/18)	−21,−94,−2	50	4.15	0.017*
	L DLPFC	−51, 26, 31	55	3.89	0.034*
	DMPFC	−6, 14, 49	8	3.67	0.063$
	R anterior insula	33, 17, 1	5	3.27	>0.10
Main effect time	NS				
Main effect condition	NS				
Time×condition interaction	NS^a^				
Post-hoc test: effect for time per condition					
Pre- vs. Post-“recall with EMs”	NS				
Pre- vs. Post-“recall-only”	NS				
Post-hoc test: effect for condition per time					
Pre-“recall with EMs” vs. Pre-“recall-only”	NS				
Post-“recall with EMs”	R amygdala	30, 2,−26	10	4.42	0.007*
<Post-“recall-only”(see Fig. 1)	Rostral ACC	−6, 44,−2	7	3.58	>0.10

ACC, anterior cingulate cortex; DLPFC, dorsolateral prefrontal cortex; DMPFC, dorsomedial prefrontal cortex; EM, eye movements; FWE, family-wise-error; NS, not significant.

a R amygdala: MNI=33, 2, −26; *k*=1; *z*=2.61.

* *p*-FWE-corrected<0.05;

** *p*-FWE-corrected<0.001;

$ *p*-FWE-corrected<0.10 (trend).

### Connectivity analyses (gPPI)

Across all four conditions (both pre- and post-“recall with EMs” and “recall-only”) the left amygdala showed mainly functional connectivity with the right visual cortex (see Table 3). There was no significant main effect of Time or Condition and no significant Time×Condition interaction.

**Table 3:** Exploratory functional connectivity analyses (gPPI) from seeds in the left and right amygdala to the ROIs (bilateral DLPFC, rostral ACC, DMPFC, anterior insula, and visual (association) cortex), before and after “recall with EMs” and “recall-only” conditions (at *p*-uncorrected<0.001)

	L amygdala seed				R amygdala seed			
		
	Region	MNI	*k*	*Z*	Region	MNI	*k*	*Z*
Main task effect								
All conditions	R visual cortex	12,−97,10	6	3.55	L visual cortex	−12,−94,−2	7	4.03*
					L amygdala	−21,−7,−14	7	3.16
Main effect time	NS				NS			
Main effect condition	NS				NS			
Time×condition interaction	NS				NS^a^			
Post-hoc test: effect for time per condition								
Pre- to post- “recall with EMs”	NS				NS			
Pre- > post- “recall-only”	NS				R visual cortex	12,−88, 10	6	3.55
Post-hoc test: effect for condition per time								
Pre-“recall with EMs”< Pre-“recall-only”	NS				NS			
Post-“recall with EMs”< Post-“recall-only” (see Fig. 2)	NS				Rostral ACC	6, 41, 4	9	3.77$

ACC, anterior cingulate cortex; DLPFC, dorsolateral prefrontal cortex; EM, eye movements; FWE, family wise error corrected for multiple comparisons; gPPI, generalized psychophysiological Interactions; L, left; R, right.

a Rostral ACC: MNI=−15, 41, −2; *k*=2; *z*=3.11.

* *p*-FWE-corrected<0.05;

$ *p*-FWE-corrected<0.10 (trend).

**Fig 2:**
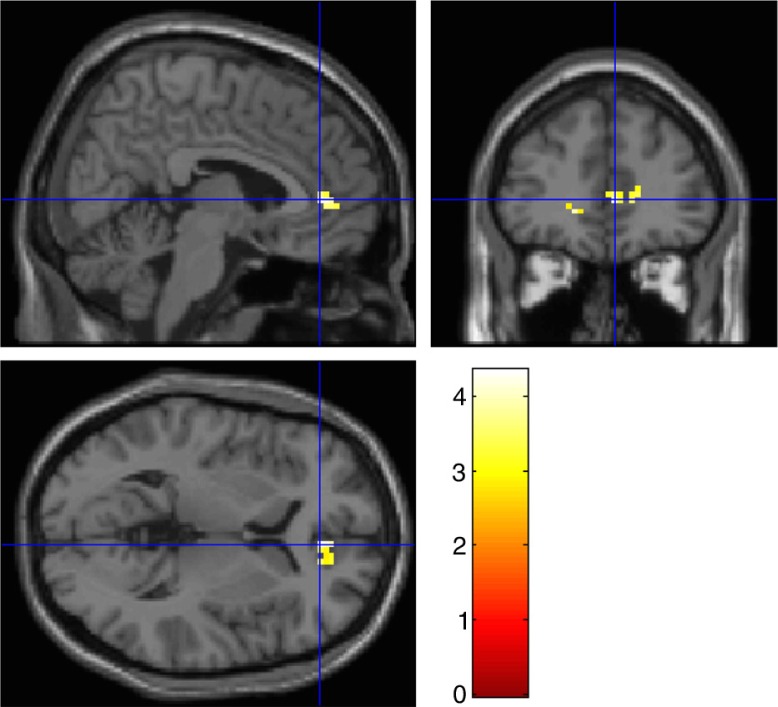
Exploratory functional connectivity analyses (gPPI) from the right amygdala seed to the ROIs (bilateral DLPFC, rostral ACC, DMPFC, anterior insula, and visual (association) cortex) (see Table 3), showing that response of the right amygdala was less correlated with rostral ACC activity in “recall with EMs” compared with the “recall-only” condition. Image was set at a threshold of *p*-uncorrected<0.005 for multiple comparisons for illustrative purposes.

The right amygdala showed mainly functional connectivity over all four conditions with the left visual cortex and the left amygdala. Functional connectivity from the right amygdala to the visual cortex decreased from pre- to post-“recall-only.” There was a—although not significant—Time × Condition interaction, indicating that functional connectivity from the right amygdala to the rostral ACC was decreased post-“recall with EMs” compared with post-“recall-only” (Fig. 2).

## Discussion and conclusions

To the best of our knowledge, this is the first fMRI study in PTSD patients investigating changes in brain activation during recall of traumatic memories with simultaneous EMs, which is part of the regular EMDR treatment protocol. Using a randomized, controlled, crossover experimental design in a small sample of eight adult patients with a primary diagnosis of PTSD, we found that the SDI protocol mainly activated the visual (association) cortex (BA 17/18), emotional processing-related (anterior insula, rostral ACC, and DMPFC) and WM-related brain regions (DLPFC). Although the predicted pre- to post-test decrease in amygdala activation after “recall with EMs” was not significant, amygdala activity was lower after “recall with EMs” than post-“recall-only.” After “recall with EMs,” rostral ACC activity was also lower compared to post-“recall-only,” but there were no significant differences in the DLPFC between conditions. Furthermore, both amygdala showed mainly functional connectivity to the visual cortex, and the right amygdala-rostral-ACC connectivity was decreased after “recall with EMs” compared with “recall-only.”

Decreased activity in the temporal lobe has consistently been found after trauma-focused psychotherapy (Lindauer et al., 2008; Pagani et al., 2007, for a review see Thomaes et al., 2014). Specifically, decreased amygdala activity has been found after individual trauma-focused psychotherapy (Felmingham et al., 2007; Peres et al., 2007, 2011) and decreased insula activity after individual and group trauma-focused cognitive (behavioral) treatment (Peres et al., 2007, 2011; Thomaes et al., 2012). However, our findings are at odds with the findings from a previous study in healthy controls, showing that bilateral alternating stimulation resulted in *increased* amygdala activity and decreased DLPFC activity (Herkt et al., 2014). The difference with our study, apart from the study population, is that Herkt et al. (2014) used general disgusting visual stimuli instead of personal traumatic audio-scripts. Furthermore, they used auditory bilateral stimulation that has been found to be less effective than visual bilateral stimulation in reducing vividness and emotional intensity of the aversive memory (Van den Hout et al., 2011, 2012).

Amygdala-rostral-ACC connectivity decreased after “recall with EMs” compared with “recall-only.” Rostral ACC activity is associated with increased emotional awareness and serves as a central hub for cognitive and emotional networks (Gröne et al., 2015). The role of the ACC in self-regulatory processes has also been measured with real-time fMRI during neurofeedback (see for a meta-analysis Emmert et al., 2016). This might implicate that “recall-only” is more disturbing, because after “recall-only” there is still more emotional processing going on via the rostral ACC, while decreased amygdalo-visual connectivity from pre- to post-“recall-only” indicates that this is at the expense of input from the visual cortex.

\EMDR consists of a full package, and making EMs during recall is just part of it. Experimental lab studies have provided strong support for a WM theory (see Van den Hout & Engelhard, 2012), but we found that the DLPFC was activated in both “recall with EMs” and “recall-only” conditions. DLPFC activity might suggest associated WM activation, but the DLPFC is also associated with emotion regulation, specifically with re-appraisal (see Zilverstand, Parvaz, & Goldstein, 2016). Because the expected Time × Condition interaction was not found, these preliminary data do not provide further evidence to support the WM model. This study indicates apparent differences in emotion processing areas other than the DLPFC between “recall with EMs” and “recall-only.”

### Limitations

The main limitation was that the study is underpowered, due to low inclusion rates related to both patient characteristics and organizational circumstances. We lost data of two participants due to feelings of panic leading to interruption of the scan session, and data of three participants due to moving artifacts, perhaps because SDI combined with the EM intervention increased emotional instability and intense emotions, while lying in a scan apparatus. Finally, the interplay between the apparatus and software (E-prime, eye-tracker) lead to the loss of two scans. The experimental set-up of this study was innovative and technically challenging. We were not able to establish the relationship between task-related brain activity patterns and subjective measures, perhaps because of the small sample size. The results warrant replication in a larger and independent sample.

## Conclusion

These preliminary results provide further support for beneficial effects of performing a dual task such as EMs during traumatic memory retrieval. Although this study focused on the EM component of EMDR, future studies may compare intervention-induced changes in brain network function across different treatments for PTSD. Future well-powered studies may also translate this information on exposure-induced network modulation to the prediction treatment outcome and disease prognosis. Previous research showed that in a specific group of PTSD patients—complex PTSD after multiple child abuse experiences—the amygdala was not activated before treatment (Thomaes et al., 2014) and this might be responsible for the lower response rates of this specific group to EMDR and other trauma-focused treatments (Dorrepaal et al., 2014). More insight in the specific neural characteristics of patients related to disease profile and disease stage and underlying mechanisms of action may lead to personalized treatment allocation and increased therapeutic efficiency (Van den Heuvel, 2015).

## References

[CIT0001] Baddeley A.D, Andrade J (2000). Working memory and the vividness of imagery. Journal of Experimental Psychology: General.

[CIT0002] Barrowcliff A.L, Gray N.S, MacCulloch S, Freeman T.C, MacCulloch M.J (2003). Horizontal rhythmical eye movements consistently diminish the arousal provoked by auditory stimuli. British Journal of Clinical Psychology.

[CIT0003] Beck A.T, Steer R.A, Garbin M.G (1998). Psychometric properties of the Beck Depression Inventory. Clinical Psychology Review.

[CIT0004] Boe H.J, Holgersen K.H, Holen A (2010). Reactivation of posttraumatic stress in male disaster survivors: The role of residual symptoms. Journal of Anxiety Disorders.

[CIT0006] Bremner J.D, Krystal J.H, Putnam F.W, Southwick S.M, Marmar C, Charney D.S, Mazure C.M (1998). Measurement of dissociative states with the Clinician-Administered Dissociative States Scale (CADSS). Journal of Traumatic Stress.

[CIT0007] Breslau N, Kessler R.C, Chilcoat H.D, Schultz L.R, Davis G.C, Andreski P (1998). Trauma and posttraumatic stress disorder in the community: The 1996 Detroit Area Survey of Trauma. Archives of General Psychiatry.

[CIT0008] Cox R.W (1996). AFNI: Software for analysis and visualization of functional magnetic resonance neuroimages. Computers and Biomedical Research.

[CIT0009] Curtis C.E, D'Esposito M (2003). Persistent activity in the prefrontal cortex during working memory. Trends in Cognitive Science.

[CIT0011] Dorrepaal E, Thomaes K, Van Balkom A.J.L.M, Veltman D.J, Hoogendoorn A.W, Draijer N (2014). Evidence based treatment for adult women with child abuse related Complex PTSD: A quantitative review. European Journal of Psychotraumatology.

[CIT0012] Elofsson U.O, Von Schèele B, Theorell T, Söndergaard H.P (2008). Physiological correlates of eye movement desensitization and reprocessing. Journal of Anxiety Disorders.

[CIT0013] Emmert K, Kopel R, Sulzer J, Brühl A.B, Berman B.D, Linden D.E, Haller S, … (2016). Meta-analysis of real-time fMRI neurofeedback studies using individual participant data: How is brain regulation mediated?. Neuroimage.

[CIT0014] Engelhard I.M, Arntz A, Van den Hout M.A (2007). Low specificity of symptoms on the post-traumatic stress disorder (PTSD) symptom scale: A comparison of individuals with PTSD, individuals with other anxiety disorders and individuals without psychopathology. British Journal of Clinical Psychology.

[CIT0015] Engelhard I.M, Van den Hout M.A, Smeets M.A (2011). Taxing working memory reduces vividness and emotional intensity of images about the Queen's Day tragedy. Journal of Behavior Therapy and Experimental Psychiatry.

[CIT0016] Engelhard I.M, Van Uijen S.L, Van den Hout M.A (2010). The impact of taxing working memory on negative and positive memories. European Journal of Psychotraumatology.

[CIT0018] Felmingham K, Kemp A, Williams L, Das P, Hughes G, Peduto A, Bryant R (2007). Changes in anterior cingulate and amygdala after cognitive behavior therapy of posttraumatic stress disorder. Psychological Science.

[CIT0019] First M.B, Spitzer R.L, Williams J.B.W, Gibbon M (1995). Structured Clinical Interview for DSM-IV Diagnoses (SCID-I).

[CIT0020] Foa E.B, Riggs D.S, Dancu C.V, Rothbaum B.O (1993). Reliability and validity of a brief instrument for assessing posttraumatic stress disorder. Journal of Traumatic Stress.

[CIT0021] Ganis G, Thompson W.L, Kosslyn S.M (2004). Brain areas underlying visual mental imagery and visual perception: An fMRI study. Cognitive Brain Research.

[CIT0022] Gröne M, Dyck M, Koush Y, Bergert S, Mathiak K.A, Alawi E.M, Mathiak K, … (2015). Upregulation of the rostral anterior cingulate cortex can alter the perception of emotions: fMRI-based neurofeedback at 3 and 7 T. Brain Topography.

[CIT0023] Gunter R.W, Bodner G.E (2008). How eye movements affect unpleasant memories: Support for a working-memory account. Behaviour Research and Therapy.

[CIT0024] Herkt D, Tumani V, Grön G, Kammer T, Hofmann A, Abler B (2014). Facilitating access to emotions: Neural signature of EMDR stimulation. PLoS One.

[CIT0025] Kearns M, Engelhard I.M (2015). Psychophysiological responsivity to script-driven imagery: An exploratory study of the effects of eye movements on public speaking flashforwards. Frontiers in Psychiatry.

[CIT0026] Kosslyn S.M, Ganis G, Thompson W.L (2001). Neural foundations of imagery. Nature Reviews Neuroscience.

[CIT0027] Lamprecht F, Köhnke C, Lempa W, Sack M, Matzke M, Münte T.F (2004). Event-related potentials and EMDR treatment of post-traumatic stress disorder. Neuroscience Research.

[CIT0028] Lanius R.A, Williamson P.C, Boksman K, Densmore M, Gupta M, Neufeld R.W, Menon R.S (2002). Brain activation during script-driven imagery induced dissociative responses in PTSD: A functional magnetic resonance imaging investigation. Biological Psychiatry.

[CIT0029] Lee C.W, Cuijpers P (2013). A meta-analysis of the contribution of eye movements in processing emotional memories. Journal of Behavior Therapy and Experimental Psychiatry.

[CIT0030] Lindauer R.J, Booij J, Habraken J.B, Van Meijel E.P, Uylings H.B, Olff M, Gersons B.P, … (2008). Effects of psychotherapy on regional cerebral blood flow during trauma imagery in patients with posttraumatic stress disorder: A randomized clinical trial. Psychological Medicine.

[CIT0031] McLaren D.G, Ries M.L, Xu G, Johnson S.C (2012). A generalized form of context-dependent psychophysiological interactions (gPPI): A comparison to standard approaches. NeuroImage.

[CIT0032] Orr S.P, Roth W.T (2000). Psychophysiological assessment: Clinical applications for PTSD. Journal of Affective Disorders.

[CIT0033] Owen A.M, McMillan K.M, Laird A.R, Bullmore E (2005). N-back working memory paradigm: A meta-analysis of normative functional neuroimaging studies. Human Brain Mapping.

[CIT0034] Pagani M, Di Lorenzo G, Monaco L, Daverio A, Giannoudas I, La Porta P, Siracusano A, … (2015). Neurobiological response to EMDR therapy in clients with different psychological traumas. Frontiers of Psychology.

[CIT0035] Pagani M, Hogberg G, Salmaso D, Nardo D, Sundin O, Jonsson C, Hällström T, … (2007). Effects of EMDR psychotherapy on 99mTc-HMPAO distribution in occupation-related post-traumatic stress disorder. Nuclear Medicine Communications.

[CIT0036] Peres J.F, Foerster B, Santana L.G, Fereira M.D, Nasello A.G, Savoia M, Lederman H, … (2011). Police officers under attack: Resilience implications of an fMRI study. Journal Psychiatry Research.

[CIT0037] Peres J.F, Newberg A.B, Mercante J.P, Simao M, Albuquerque V.E, Peres M.J, Nasello A.G (2007). Cerebral blood flow changes during retrieval of traumatic memories before and after psychotherapy: A SPECT study. Psychological Medicine.

[CIT0038] Pitman R.K, Orr S.P, Forgue D.F, De Jong J.B, Claiborn J.M (1987). Psychophysiologic assessment of posttraumatic stress disorder imagery in Vietnam combat veterans. Archives of General Psychiatry.

[CIT0039] Propper R.E, Pierce J, Geisler M.W, Christman S.D, Bellorado N (2007). Effect of bilateral eye movements on frontal interhemispheric gamma EEG coherence: Implications for EMDR therapy. Journal of Nervous and Mental Disease.

[CIT0040] Rabinak C.A, MacNamara A, Kennedy A.E, Angstadt M, Stein M.B, Liberzon I, Phan K.L (2014). Focal and aberrant prefrontal engagement during emotion regulation in veterans with posttraumatic stress disorder. Depression and Anxiety.

[CIT0041] Rauch S.L, Shin L.M, Phelps E.A (2006). Neurocircuitry models of posttraumatic stress disorder and extinction: Human neuroimaging research—Past, present, and future. Biological Psychiatry.

[CIT0042] Resick P.A, Nishith P, Weaver T.L, Astin M.C, Feuer C.A (2002). A comparison of cognitive-processing therapy with prolonged exposure and a waiting condition for the treatment of chronic posttraumatic stress disorder in female rape victims. Journal of Consulting and Clinical Psychology.

[CIT0043] Rottschy C, Langner R, Dogan I, Reetz K, Laird A.R, Schulz J.B, Eickhoff S.B, … (2012). Modelling neural correlates of working memory: A coordinate-based meta-analysis. NeuroImage.

[CIT0044] Sack M, Lempa W, Steinmetz A, Lamprecht F, Hofmann A (2008). Alterations in autonomic tone during trauma exposure using eye movement desensitization and reprocessing (EMDR)—Results of a preliminary investigation. Journal of Anxiety Disorders.

[CIT0045] Samara Z, Elzinga B.M, Slagter H.A, Nieuwenhuis S (2011). Do horizontal saccadic eye movements increase interhemispheric coherence? Investigation of a hypothesized neural mechanism underlying EMDR. Frontiers of Psychiatry.

[CIT0046] Schubert S.J, Lee C.W, Drummond P.D (2011). The efficacy and psychophysiological correlates of dual-attention tasks in eye movement desensitization and reprocessing (EMDR). Journal of Anxiety Disorders.

[CIT0060] Shapiro F (1989). Eye movement desensitization: a new treatment for post-traumatic stress disorder. J Behav Ther Exp Psychiatry.

[CIT0047] Shin L.M, Orr S.P, Carson M.A, Rauch S.L, Macklin M.L, Lasko N.B, Pitman R.K (2004). Regional cerebral blood flow in the amygdala and medial prefrontal cortex during traumatic imagery in male and female Vietnam veterans with PTSD. Archives of General Psychiatry.

[CIT0048] Spielberger C.D (1983). Manual for the State-Trait Anxiety Inventory (STAI).

[CIT0049] Thomaes K, Dorrepaal E, Draijer N, De Ruiter M.B, Elzinga B.M, Van Balkom A.J, Veltman D.J, … (2012). Treatment effects on insular and anterior cingulate cortex activation during classic and emotional Stroop interference in child abuse-related complex post-traumatic stress disorder. Psychological Medicine.

[CIT0050] Thomaes K, Dorrepaal E, Draijer N, Jansma E.P, Veltman D.J, Van Balkom A.J (2014). Can pharmacological and psychological treatment change brain structure and function in PTSD?. A systematic review. Journal of Psychiatric Research.

[CIT0051] Trentini C, Pagani M, Fania P, Speranza A.M, Nicolais G, Sibilia A, Ammaniti M, … (2015). Neural processing of emotions in traumatized children treated with Eye Movement Desensitization and Reprocessing therapy: A hdEEG study. Frontiers of Psychology.

[CIT0052] Van Balkom A.L.J.M, Van Vliet I.M, Emmelkamp P.M.G, Bockting C.L.H, Spijker J, Hermens M.L.M, Meeuwissen J.A.C, namens de Werkgroep Multidisciplinaire richtlijnontwikkeling Angststoornissen/Depressie (2013). Multidisciplinaire richtlijn Angststoornissen (Derde revisie). Richtlijn voor de diagnostiek, behandeling en begeleiding van volwassen patiënten met een angststoornis.

[CIT0053] Van den Heuvel O.A Toward brain-based guidance of clinical practice. JAMA Psychiatry.

[CIT0054] Van den Hout M.A, Engelhard I.M, Rijkeboer M.M, Koekebakker J, Hornsveld H, Leer A, Akse N, … (2011). EMDR: Eye movements superior to beeps in taxing working memory and reducing vividness of recollections. Behaviour Research and Therapy.

[CIT0059] Van den Hout M.A, Engelhard I.M (2012). How does EMDR work?. Journal of Experimental Psychopathology.

[CIT0055] Van den Hout M.A, Rijkeboer M.M, Engelhard I.M, Klugkist I, Hornsveld H, Toffolo M.J, Cath D.C (2012). Tones inferior to eye movements in the EMDR treatment of PTSD. Behaviour Research and Therapy.

[CIT0056] Van der Ploeg H.M, Defares P.B, Spielberger C.D (1980). Handleiding bij de Zelf-Beoordelings Vragenlijst, ZBV. Een Nederlandstalige bewerking van de Spielberger State-Trait Anxiety Inventory, STAT-DY.

[CIT0057] World Health Organization (2013). Guidelines for the management of conditions specifically related to stress.

[CIT0058] Zilverstand A, Parvaz M.A, Goldstein R.Z (2016). Neuroimaging cognitive reappraisal in clinical populations to define neural targets for enhancing emotion regulation. A systematic review. Neuroimage.

